# Stigma in first epizode patients with schizophrenia

**DOI:** 10.1192/j.eurpsy.2024.1586

**Published:** 2024-08-27

**Authors:** B. Aukst Margetić, B. Margetić, M. Vilibić

**Affiliations:** ^1^Department of psychiatry, University hospital center Sestre milosrdnice; ^2^ Catholic University of Croatia; ^3^Department of psychiatry, University hospital Dubrava, Zagreb, Croatia

## Abstract

**Introduction:**

Patients with schizophrenia confront with stigmatization in their everyday life. Differences in their perception of stigmatization based on the number of hospitalizations and duration of treatment are unsufficiently researched.

**Objectives:**

Our aim was to investigate whether patients with first-episode schizophrenia differ in their perception of stigmatization from schizophrenia patients with more than one hospitalization,

**Methods:**

A consecutive sample of 120 stable outpatients (70 males, 50 female) diagnosed with schizophrenia were included in the study. Diagnosis of schizophrenia was established with Neuropsychiatric Interview. First episode patients consisted 28.3% of the group.

All patients were at least once hospitalized for mental illness. Patients were dichotomised based on the number of hospitalizations.

The study was approved by Ethic committee of the institutions. Stigma was assessed with Internalized Stigma of Mental Illness (ISMI) scale.

ISMI scale contains 29 Likert items rated on a 4-point scale ranging from “strongly disagree” to “strongly agree”. It contains five subscales: Alienation, Stereotype Endorsement, Discrimination Experience, Social Withdrawal and Stigma Resistance. The overall internal consistency for the global ISMI was 0,89; Alienation-0,76; Stereotype endorsement- 0,63; Discrimination- 0,72; Social withdrawal- 0,57.

All analyses were performed using the SPSS 25.0. The differences between groups on continuous variables were evaluated using t-test with Bonferroni correction. For all analyses, the level of statistical significance was defined as an alpha less than 0.05

**Results:**

There were no differences in first-episode and more episode patietns in ISMI and its subscales. Number of hospitalizations was associated with Stereotype endorsement subscale (r=228;p=0,012) Age was correlated with stigma.

**Conclusions:**

Although stigma did not differ between first-episode patients and patients with two or more hospitalizations, stereotype endorsement was strongy associated with the number of hospitalizations leading to conclusion that stigma is associated with psychiatric treatment and our aim must be to destigmatize the treatment and avoid hospitalizations.

**Disclosure of Interest:**

None Declared

